# Theory and development of biplanar active shim coils for a permanent NMR analyzer

**DOI:** 10.1371/journal.pone.0181552

**Published:** 2017-07-24

**Authors:** Tian Xia, Zhiying Miao, Shanshan Chen, Hongzhi Wang, Yefeng Yao

**Affiliations:** 1 Shanghai Key Laboratory of magnetic resonance, East China Normal University, Shanghai, P. R. China; 2 Medical Imaging College, Shanghai University of Medicine and Health Science, Shanghai, P.R. China; 3 School of Optical- Electrical and Computer Engineering, University of Shanghai for Science and Technology Shanghai, P.R. China; Universidad Miguel Hernandez de Elche, SPAIN

## Abstract

The general expression for magnetic induction in the z axis direction is derived from magnetic scalar potential, and magnetic induction for biplanar shimming coils (BSCs) is also derived from magnetic vector potentials and Green functions, which simultaneously include Sin and Cos harmonic fields. The relationship between these expressions is discussed, and we show they are partially consistent. Magnetic induction generated Sin and Cos stream functions, which are presented and discussed, and we conclude that the type of stream function determines the type of harmonic field, and that BSCs can not only generate specific harmonic fields directly using Cos stream function, but also generate the rest of the harmonic fields through some specific operations. The detailed design process is presented in the form of a diagram. Subsequently, nine BSCs were calculated using the proposed method and applied to a low field NMR relaxation analyzer. The magnetic field homogeneity after shimming increases significantly, which verifies its practical value.

## Introduction

Low field NMR (LF-NMR) relaxation analysis technics has been widely employed in oil exploration, food safety, life sciences, high molecular material, and mineral engineering since it was developed in the 1980s[1–5]. LF-NMR has advantages in running costs, and it is non-invasive, dependable, and highly secure, and it will be expanded to new applications as portability and miniaturization develops. However, it also has disadvantages relative to high field NMR, such as low resolution and low sensitivity due to the magnetic field inhomogeneity, which has shown LF-NMR to be insufficient in detecting small composition content, and it has a short relaxation time.

The primary requirement of any NMR system is that its main magnetic field must be highly uniform. LF-NMR usually adopts rare earth permanent magnets to generate the main magnetic field, which generally has significantly lower homogeneity than that generated by superconducting magnets. Two methods are used to improve field homogeneity: passive and active shimming. Passive shimming is the basic method, with active shimming adjusting the field further to attain improved homogeneity. Passive shimming must be carried out in advance because the initial coil current using just active shimming may be so high that the generated heat will affect the magnet temperature stability. This paper investigates active shim coil design to improve homogeneity based on passive shimming.

Target field is a well-known design method proposed by Turner[6] in 1986. This method has no ill-conditioned problem because Fourier transforms have unique inverses, and it has been used to design cylindrical surface shim coils for superconducting magnets for magnetic resonance imaging (MRI). Forbes and Crozier[7–9] advanced the TF method, considering the finite coil length using a Fourier series technique. Wen Tao Liu[10–12] proposed an alternative current density expansion, rather than Tikhonov regularization, and Xiao Fei You[13] combined this method with Forbes and Crozier’s. Ge Li Hu et al.[14,15] employed L1 norm regularization to replace the original ill-conditioned problem with a nearly well-conditioned problem. There has been considerable TF research, and application of TF in engineering has expanded rapidly. However, shim coils based on TF focus on superconducting magnetic resonance spectroscopy (SMRS) to gain high homogeneity for most research, which generally has a small region of interest (ROI), approximately Φ × H = 5 mm × 20 mm. This paper proposes a biplanar shim coils (BSCs) approach for LF-NMR to improve homogeneity to 0.5 ppm over a relative large ROI (Φ × H = 25.4 mm × 40 mm). Most literature has only simulated results to demonstrate the efficiency of their proposed approach, whereas this paper uses physical experiments to validate the proposed approach.

The general expression for magnetic induction from a magnetic scalar potential and the specific magnetic induction for BSCs are derived, and their relationship discussed. We present a more general methodology, valid for spherical harmonics that includes Sin and Cos functions simultaneously. We demonstrate that the BSCs not only directly generate some specific harmonic fields, but also generate all other harmonic fields through some specific operations. The detailed design process is shown diagrammatically, and nine BSC pairs are designed based on the indicated process, then installed in a biplanar permanent MRI. An experiment was performed to verify the shimming effect, with very significant results, which indicates that the theory and development approach is viable.

## Theory

### General formula

The main field, ***B***_**0**_, is usually in the z axis direction, which is perpendicular to the radio frequency (RF) direction. In diameter spherical volume (DSV), consider a small deviation of ***B***_**0**_, ***B****'* ≪ ***B***_**0**_, where the total field is

$$B=B_{0}+B^{\prime }.$$ (1)

Since RF is perpendicular to ***B***_**0**_, only ***B*'** in the z axis direction affects the NMR signal, so

$$B_{z}=B_{0}+{B^{\prime }}_{Z}$$ (2)

in the DSV through which no current passes for a static magnetic field, so that **∇** × ***B*** = 0 from Maxwell's equations. It then follows from a vector identity that

$$B=-\mu_{0}\nabla \Psi ,$$ (3)

where *Ψ* and *μ*_0_ are the magnetic scalar potential and magnetic conductivity in a vacuum, respectively. Maxwell’s equations require **∇** ⋅ ***B*** = 0, which means *Ψ* satisfies the Laplace equation in air in DSV, i.e.,

$$\nabla^{2}\Psi =0$$ (4)

**Fig 1:**
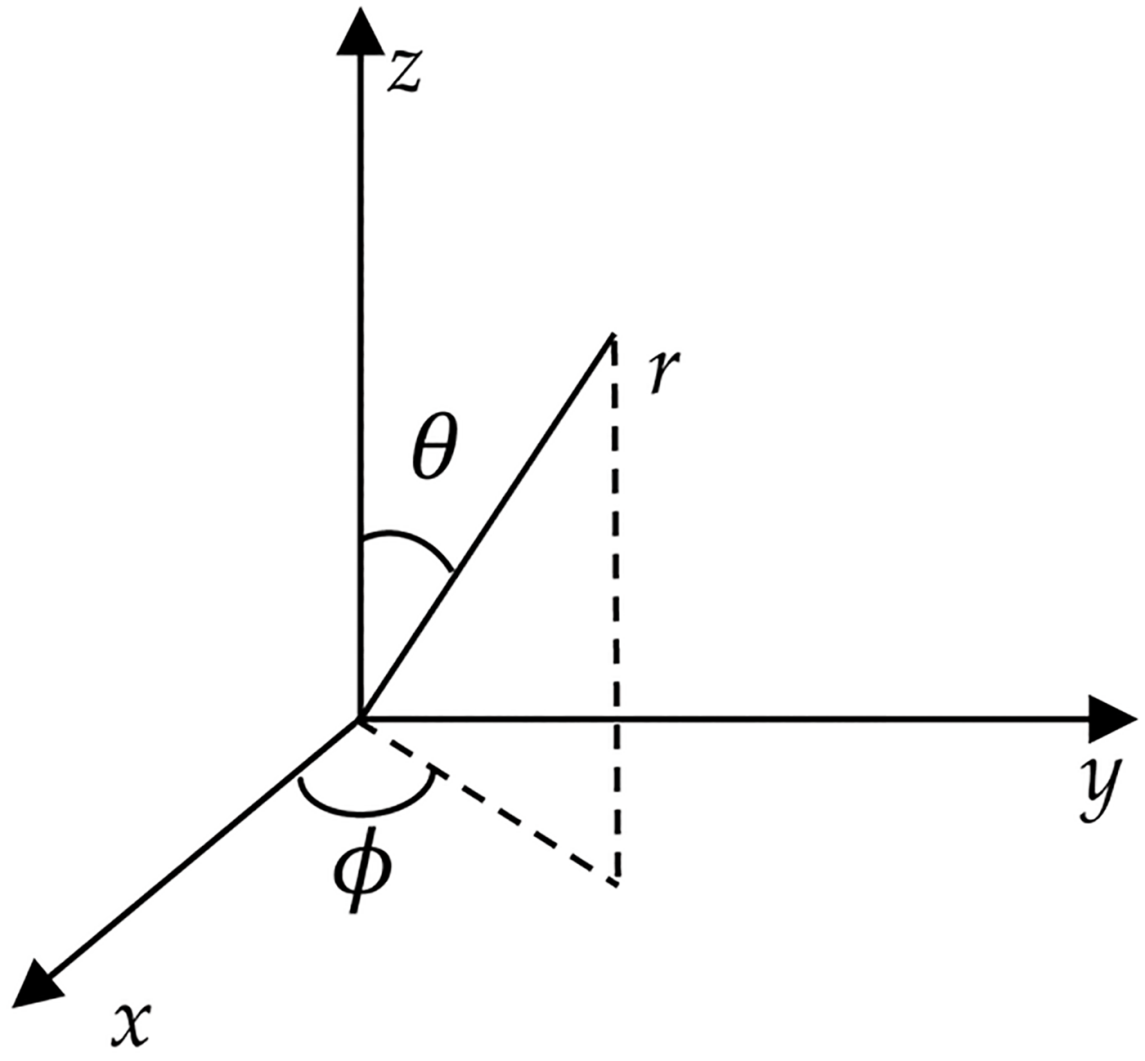
Spherical and rectangular coordinates.

Eq (4) can be solved in spherical polar coordinates rather than Cartesian coordinates, using the usual transformation relations between the coordinate systems, which are presented here explicitly for clarify (Fig 1),

x=r sinθ cosϕ, y=r sinθ sinϕ, z=r cosθ, (5)

and the general solution in spherical coordinates can be expressed as

$$\Psi \left(r,\theta ,\phi \right)=\Sigma_{n=0}^{\infty }\Sigma_{m=0}^{n}r^{n}P_{n}^{m}(\text{cos}\theta )(A_{n}^{m}\text{cos}m\phi +B_{n}^{m}\text{sin}m\phi ),$$ (6)

where *n* and *m* are the degree and order of *Ψ*, respectively; $P_{n}^{m}$ is an associated Legendre function; and $A_{m}^{n}$,$\;B_{n}^{m}$ are coefficients independent of *r*,*θ*, and *ϕ*. When *n* = 0, then *m* = 0 also, from Eq (6), and *Ψ* is constant.

From the gradient transformation,

$$B_{z}=-\mu_{0}\frac{\partial \Psi }{\partial z}=\mu_{0}\left(-\text{cos}\theta \frac{\partial \Psi }{\partial r}+\frac{\text{sin}\theta }{r}\frac{\partial \Psi }{\partial \theta }\right).$$ (7)

Substituting Eqs (6) into (7),

$$B_{z}=\mu_{0}\{\Sigma_{n=1}^{\infty }\Sigma_{m=0}^{n}r^{n-1}[\left(2n+1\right)\text{cos}\theta P_{n}^{m}\left(\text{cos}\theta \right)-\left(n-m+1\right)P_{n+1}^{m}\left(\text{cos}\theta \right)\text{]}\times (A_{n}^{m}\text{cos}m\phi +B_{n}^{m}\text{sin}m\phi )\},$$

which can be simplified using the properties of Legendre functions as

$$B_{z}=\mu_{0}\Sigma_{n=1}^{\infty }\Sigma_{m=0}^{n}r^{n-1}\left(n+m\right)P_{n-1}^{m}\left(\text{cos}\theta \right)\times (A_{n}^{m}\text{cos}m\phi +B_{n}^{m}\text{sin}m\phi ).$$ (8)

Since *m* ≤ *n* for $P_{n}^{m}\left(x\right)$ for Legendre functions, then *m* ≤ *n* −1 for $P_{n-1}^{m}\left(\text{cos}\theta \right)$ in Eq (8), so

$$B_{z}=\mu_{0}\Sigma_{n=1}^{\infty }\Sigma_{m=0}^{n-1}r^{n-1}\left(n+m\right)P_{n-1}^{m}\left(\text{cos}\theta \right)\times (A_{n}^{m}\text{cos}m\phi +B_{n}^{m}\text{sin}m\phi ),$$ (9)

and for convenience, let *α* = n − 1 and *β* = *m*, then

$$\begin{array}{ll} B_{z} & =\mu_{0}\Sigma_{\alpha =0}^{\infty }\Sigma_{\beta =0}^{\alpha }\left(\alpha +\beta +1\right)r^{\alpha }P_{\alpha }^{\beta }\left(\text{cos}\theta \right)\times (A_{\alpha +1}^{\beta }\text{cos}\beta \phi +B_{\alpha +1}^{\beta }\text{sin}\beta \phi )\\ & =\mu_{0}\Sigma_{\alpha =0}^{\infty }\Sigma_{\beta =0}^{\alpha }\left(\alpha +\beta +1\right)r^{\alpha }P_{\alpha }^{\beta }\left(\text{cos}\theta \right){\left(\begin{array}{c} A_{\alpha +1}^{\beta }\\ B_{\alpha +1}^{\beta } \end{array}\right)}^{T}\left(\begin{array}{c} \text{cos}\beta \phi \\ \text{sin}\beta \phi \end{array}\right), \end{array}$$ (10)

where *α* and *β* here are the degree and order of *B*_*z*_.

Eq (10) is the general expression derived from *Ψ*, and is compared to the BSC derived expression in the following section. This indicates that *B*_*z*_ is composed of a series of harmonic fields, which is consistent with Anderson’s theory[6]. In the ideal case, Eq (10) should contain only the uniform field, *B*_*z*0_, and the constant $A_{1}^{0}$ with no higher harmonics present when *α* = *β = 0*. For given *α* ≠ 0 and *β* ≠ 0, $r^{\alpha }P_{\alpha }^{\beta }\left(\text{cos}\theta \right)\left(\text{cos}\beta \phi ¦\text{sin}\beta \phi \right)$ determines the distribution of *B*_*z*_’s higher harmonic. The purpose of shim coils is to eliminate higher harmonics and keep the field uniform. Hence, the target field should be chosen from Table 1.

**Table 1:** Harmonic field expressions for *B*_*z*_ in rectangular and spherical coordinates.

Degree (*α*)	Order (*β*)	Abbreviation	Coefficients	Spherical coordinates	Rectangular coordinates
**0**	**0**	Z^0^	$A_{1}^{0}$	1	1
**1**	**0**	Z	$2A_{2}^{0}$	*r*cos *θ*	*z*
**1**	**1**	X	$3A_{2}^{1}$	*r*sin *θ* cos *ϕ*	*x*
**1**	**1**	Y	$3B_{2}^{1}$	*r*sin *θ* sin *ϕ*	*y*
**2**	**0**	Z^2^	$3A_{3}^{0}$	*r*^2^ (3cos^2^ *θ* − 1)/2	*z*^2^ − (*x*^2^ + *y*^2^)/2
**2**	**1**	XZ	$12A_{3}^{1}$	*r*^2^ cos *θ* sin *θ* cos *ϕ*	*xz*
**2**	**1**	YZ	$12B_{3}^{1}$	*r*^2^ cos *θ* sin *θ* sin *ϕ*	*yz*
**2**	**2**	X^2^-Y^2^	$15A_{3}^{2}$	*r*^2^ sin^2^ *θ* cos2*ϕ*	*x*^2^ − *y*^2^
**2**	**2**	2XY	$15B_{3}^{2}$	*r*^2^ sin^2^ *θ* sin2*ϕ*	2*xy*
**3**	**0**	Z^3^	$4A_{4}^{0}$	*r*^3^ cos*θ* (5cos^2^ *θ* − 3)/2	*z*[*z*^2^ − 3(*x*^2^ + *y*^2^)/2]
**3**	**1**	XZ^2^	$15A_{4}^{1}$	*r*^3^ sin*θ* cos*ϕ* (5cos^2^ *θ* − 1)/2	*x*[4*z*^2^ − (*x*^2^ + *y*^2^)]
**3**	**1**	YZ^2^	$15B_{4}^{1}$	*r*^3^ sin*θ* sin*ϕ* (5cos^2^ *θ* − 1)/2	*y*[4*z*^2^ − (*x*^2^ + *y*^2^)]
**3**	**2**	Z(X^2^-Y^2^)	$90A_{4}^{2}$	*r*^3^ cos*θ* sin^2^*θ*cos2*ϕ*	*z*(*x*^2^ − *y*^2^)
**3**	**2**	XYZ	$90B_{4}^{2}$	*r*^3^ cos*θ* sin^2^*θ*sin2*ϕ*	2*xyz*
**3**	**3**	X^3^	$105A_{4}^{3}$	*r*^3^ sin^3^*θ*cos3*ϕ*	*x*^3^ − 3*xy*^2^
**3**	**3**	Y^3^	$105B_{4}^{3}$	*r*^3^ sin^3^*θ*sin3*ϕ*	3*x*^2^y − *y*^3^
**4**	**0**	Z^4^	$5A_{5}^{0}/8$	*r*^4^(35cos^4^ *θ* − 30cos^2^ *θ* + 3)	8*z*^4^ − 24*z*^2^ (*x*^2^ + *y*^2^) +3(*x*^2^ + *y*^2^)^2^
**4**	**1**	XZ^3^	$15A_{5}^{1}$	*r*^4^ sin*θ*cos*ϕ*cos*θ*(7cos^2^*θ* − 3)	*x*[4*z*^3^ − 3(*x*^2^ + *y*^2^)*z*]
**4**	**1**	YZ^3^	$15B_{5}^{1}$	*r*^4^ sin*θ*sin*ϕ*cos*θ*(7cos^2^*θ* − 3)	*y*[4*z*^3^ − 3(*x*^2^ + *y*^2^)*z*]
**4**	**2**	Z^2^(X^2^-Y^2^)	$105A_{5}^{2}/2$	*r*^4^ sin^2^ *θ*cos2*ϕ*(7cos^2^*θ* − 1)	[6*z*^2^ − (*x*^2^ + *y*^2^)]×(*x*^2^ − *y*^2^)
**4**	**2**	XYZ^2^	$105B_{5}^{2}/2$	*r*^4^ sin^2^*θ*sin2*ϕ*(7cos^2^*θ* − 1)	[6*z*^2^ − (*x*^2^ + *y*^2^)]×2*xy*
**4**	**3**	ZX^3^	$840A_{5}^{3}$	*r*^4^ cos*θ*sin^3^*θ*cos3*ϕ*	*z*[*x*^3^ − 3*xy*^2^]
**4**	**3**	ZY^3^	$840B_{5}^{3}$	*r*^4^ cos*θ*sin^3^*θ*sin3*ϕ*	*z*[*y*^3^ − 3*yx*^2^]
**4**	**4**	(X^2^-Y^2^)^2^	$945A_{5}^{4}$	*r*^4^ sin^4^*θ*cos4*ϕ*	(*x*^2^ − *y*^2^)^2^ − 4*x*^2^*y*^2^
**4**	**4**	XY(X^2^-Y^2^)	$945B_{5}^{4}$	*r*^4^ sin^4^*θ*sin4*ϕ*	4*xy*(*x*^2^ − *y*^2^)
**5**	**0**	Z^5^	$3A_{6}^{0}/4$	*r*^5^ cos*θ*(63cos^4^*θ* − 70cos^2^*θ* + 15)	8*z*^5^ − 40*z*^3^ (*x*^2^ + *y*^2^) +15*z*(*x*^2^ + *y*^2^)^2^

### Biplanar shim coil generated magnetic field from scalar potential

**Fig 2:**
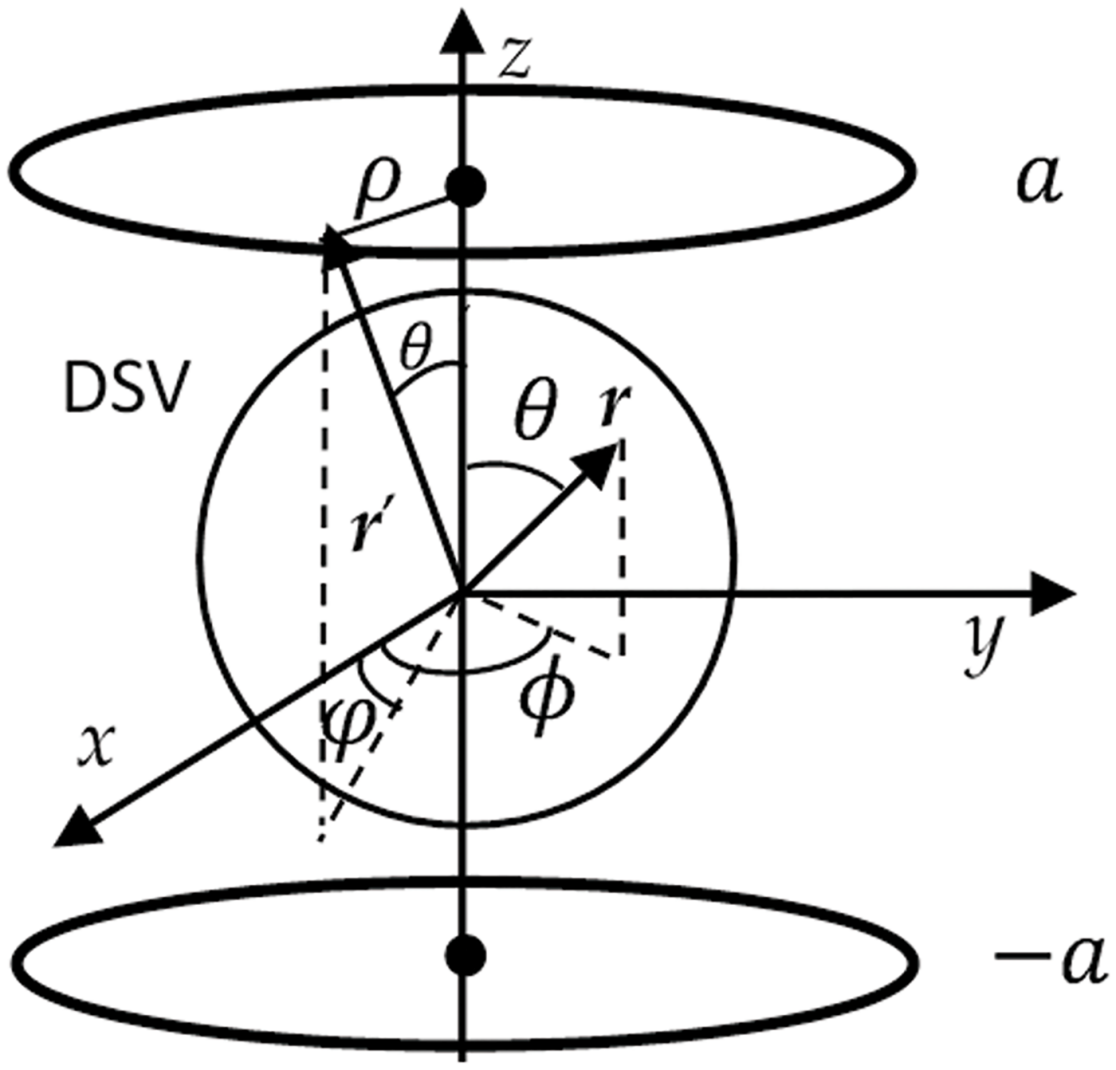
Coordinate system for biplanar shim coils.

Fig 2 shows the coordinate system for ***B***_***z***_ in DSV for BSCs, with current density located in the *z* = ±*a* planes respectively. ***r***(*r*,*θ*,*ϕ*) and ***r'***(*r'*,*θ'*,*φ*) are the position vectors of the field and source points, respectively.

According to Maxwell's equations, **∇** ⋅ ***B*** = 0. Therefore, there is a magnetic vector potential, ***A***, such that ***B* = ∇ × *A***, because **∇ × *B*** = *μ*_0_***J***, where *J* is the current density, and **∇**^2^***A* = −***μ*_0_***J***. The general solution was obtained in terms of the Green function **1**⁄|***r*** − ***r***'|, and can be expressed in differential form as

$$\text{d}A=\frac{\mu_{0}}{4\pi }\frac{1}{\left|r-r^{\prime }\right|}J\text{dV,}$$

and the differential form of the magnetic field can be expressed as

$$\text{d}B=\frac{\mu_{0}}{4\pi }\nabla \frac{1}{\left|r-r^{\prime }\right|}\times J\text{dV},$$ (11a)

However, ***J*** is planar rather than a volume distribution. Hence, Eq (11a) becomes

$$\text{d}B=\frac{\mu_{0}}{4\pi }\nabla \frac{1}{\left|r-r^{\prime }\right|}\times J\text{ds}$$ (11b)

where ds = *ρ*d*ρ*d*ϕ*. Let *G* = **1**⁄|***r*** − ***r***'| where *G* is the Green function, and define the gradient *G*' = ∇*G*, then

$$\text{d}B_{z}=\frac{\mu_{0}}{4\pi }\text{ds}(G_{x}^{\prime }J_{y}-G_{y}^{\prime }J_{x}),$$ (12)

where $G_{x}^{\prime }$ and $G_{y}^{\prime }$ are the components of ***G***' in the X and Y directions, respectively; and *J*_*x*_ and *J*_*y*_ are the components of ***J*** in the X and Y directions, respectively.

In spherical coordinates, Eq (12) can be expressed as

$$\begin{array}{ll} \text{d}B_{z} & =\frac{\mu_{0}}{4\pi }\text{ds}\left[\left(G_{r^{\prime }}^{\prime }\text{sin}\theta^{\prime }\text{cos}\varphi +G_{\theta^{\prime }}^{\prime }\text{cos}\theta^{\prime }\text{cos}\varphi -G_{\varphi }^{\prime }\text{sin}\varphi \right)\left(J_{\rho }\text{sin}\varphi +J_{\varphi }\text{cos}\varphi \right)-\left(G_{r^{\prime }}^{\prime }\text{sin}\theta^{\prime }\text{sin}\varphi +G_{\theta^{\prime }}^{\prime }\text{cos}\theta^{\prime }\text{sin}\varphi +G_{\varphi }^{\prime }\text{cos}\varphi \right)\left(J_{\rho }\text{cos}\varphi -J_{\varphi }\text{sin}\varphi \right)\right]\\ & =\frac{\mu_{0}}{4\pi }\text{ds}\left[G_{r^{\prime }}^{\prime }J_{\varphi }\text{sin}\theta^{\prime }+G_{\theta^{\prime }}^{\prime }J_{\varphi }\text{cos}\theta^{\prime }-G_{\varphi }^{\prime }J_{\rho }\right], \end{array}$$ (13)

where $G_{r^{\prime }}^{\prime }$,$G_{\theta^{\prime }}^{\prime }$, and $G_{\varphi }^{\prime }$ are the components of ***G****'* in spherical coordinates; and *J*_*ρ*_ and *J*_*φ*_ are the components of ***J*** in polar coordinates. Since $\nabla G=\frac{\partial G}{\partial r^{\prime }}{\hat{e}}_{r^{\prime }}+\frac{1}{r^{\prime }}\frac{\partial G}{\partial \theta^{\prime }}{\hat{e}}_{\theta^{\prime }}+\frac{1}{r^{\prime }\text{sin}\theta^{\prime }}\frac{\partial G}{\partial \varphi }{\hat{e}}_{\varphi }$, $G_{r^{\prime }}^{\prime }=\frac{\partial G}{\partial r^{\prime }}$, $G_{\theta^{\prime }}^{\prime }=\frac{1}{r^{\prime }}\frac{\partial G}{\partial \theta^{\prime }}$, and $G_{\varphi }^{\prime }=\frac{1}{r^{\prime }\text{sin}\theta^{\prime }}\frac{\partial G}{\partial \varphi }$. In the interval *r* ≤ a < *r'* for DSV, the Green function can be expanded[16]

$$G=1/\left|r-r^{\prime }\right|=\frac{1}{r^{\prime }}\Sigma_{\alpha =0}^{\infty }\Sigma_{\beta =0}^{\alpha }\varepsilon_{\beta }\frac{\left(\alpha -\beta \right)!}{\left(\alpha +\beta \right)!}P_{\alpha }^{\beta }\left(\text{cos}\theta^{\prime }\right){\left(\frac{r}{r^{\prime }}\right)}^{\alpha }P_{\alpha }^{\beta }\left(\text{cos}\theta \right)\text{cos}\left[\beta \left(\phi -\varphi \right)\right],$$ (14)

where $\varepsilon_{\beta }=\{\begin{array}{c} 1,\;\;\;\beta =0\\ 2,\;\;\;\beta \neq 0 \end{array}$. Substituting Eqs (14) into (13),

$$\text{d}B_{z}=\frac{\mu_{0}}{4\pi }\text{ds}\Sigma_{\alpha =0}^{\infty }\Sigma_{\beta =0}^{\alpha }\varepsilon_{\beta }\frac{\left(\alpha -\beta \right)!}{\left(\alpha +\beta \right)!}\frac{r^{\alpha }}{{r^{\prime }}^{\alpha +2}}P_{\alpha }^{\beta }\left(\text{cos}\theta \right)\times \{-\left(\alpha +1\right)J_{\varphi }\text{sin}\theta^{\prime }P_{\alpha }^{\beta }\left(\text{cos}\theta^{\prime }\right)\text{cos}\left[\beta \left(\phi -\varphi \right)\right]+J_{\varphi }\text{cos}\theta^{\prime }\frac{\partial P_{\alpha }^{\beta }\left(\text{cos}\theta^{\prime }\right)}{\partial \theta^{\prime }}\text{cos}\left[\beta \left(\phi -\varphi \right)\right]-\beta J_{\rho }\frac{P_{\alpha }^{\beta }\left(\text{cos}\theta^{\prime }\right)}{\text{sin}\theta^{\prime }}\text{sin}\left[\beta \left(\phi -\varphi \right)\right]\}.$$ (15)

From the recurrence property of associated Legendre functions[17],

$$\frac{\text{d}P_{\alpha }^{\beta }\left(\text{cos}\theta^{\prime }\right)}{\text{d}\theta^{\prime }}=\frac{1}{\text{sin}\theta^{\prime }}\left[\left(\alpha -\beta +1\right)P_{\alpha +1}^{\beta }\left(\text{cos}\theta^{\prime }\right)-\left(\alpha +1\right)\text{cos}\theta^{\prime }P_{\alpha }^{\beta }\left(\text{cos}\theta^{\prime }\right)\right],$$ (16)

then

$$\text{d}B_{z}=\frac{\mu_{0}}{4\pi }\text{ds}\sum_{\alpha =0}^{\infty }\sum_{\beta =0}^{\alpha }\frac{\left(\alpha -\beta \right)!}{\left(\alpha +\beta \right)!}\frac{r^{\alpha }}{{r^{\prime }}^{\alpha +2}}P_{\alpha }^{\beta }\left(\text{cos}\theta \right)\times \left\{\frac{1}{\text{sin}\theta^{\prime }}\left[\left(\alpha -\beta +1\right)\text{cos}\theta^{\prime }P_{\alpha +1}^{\beta }\left(\text{cos}\theta^{\prime }\right)-\left(\alpha +1\right)P_{\alpha }^{\beta }\left(\text{cos}\theta^{\prime }\right)\right]J_{\varphi }\text{cos}\left[\beta \left(\phi -\varphi \right)\right]-\beta \frac{P_{\alpha }^{\beta }\left(\text{cos}\theta^{\prime }\right)}{\text{sin}\theta^{\prime }}J_{\rho }\text{sin}\left[\beta \left(\phi -\varphi \right)\right]\right\}.$$ (17)

Let

$$C_{\alpha \beta }=\frac{\left(\alpha -\beta \right)!}{\left(\alpha +\beta \right)!}\frac{1}{{r^{\prime }}^{\alpha +2}}\text{ and }C_{\alpha \beta }^{\rho }=\beta \frac{P_{\alpha }^{\beta }\left(\text{cos}\theta^{\prime }\right)}{\text{sin}\theta^{\prime }},$$

and

$$\;C_{\alpha \beta }^{\varphi }=\frac{1}{\text{sin}\theta^{\prime }}\left[\left(\alpha -\beta +1\right)\text{cos}\theta^{\prime }P_{\alpha +1}^{\beta }\left(\text{cos}\theta^{\prime }\right)-\left(\alpha +1\right)P_{\alpha }^{\beta }\left(\text{cos}\theta^{\prime }\right)\right],$$

then Eq (17) becomes

$$\begin{array}{ll} \text{d}B_{z} & =\frac{\mu_{0}}{4\pi }\text{d}s\Sigma_{\alpha =0}^{\infty }\Sigma_{\beta =0}^{\alpha }\varepsilon_{\beta }C_{\alpha \beta }r^{\alpha }P_{\alpha }^{\beta }\left(\text{cos}\theta \right)\left\{C_{\alpha \beta }^{\varphi }J_{\varphi }\text{cos}\left[\beta \left(\phi -\varphi \right)\right]-C_{\alpha \beta }^{\rho }J_{\rho }\text{sin}\left[\beta \left(\phi -\varphi \right)\right]\right\}\\ & =\frac{\mu_{0}}{4\pi }\text{d}s\Sigma_{\alpha =0}^{\infty }\Sigma_{\beta =0}^{\alpha }\varepsilon_{\beta }C_{\alpha \beta }r^{\alpha }P_{\alpha }^{\beta }\left(\text{cos}\theta \right)[\left(C_{\alpha \beta }^{\varphi }J_{\varphi }\text{cos}\beta \varphi +C_{\alpha \beta }^{\rho }J_{\rho }\text{sin}\beta \varphi \right)\text{cos}\beta \phi +\left(C_{\alpha \beta }^{\varphi }J_{\varphi }\text{sin}\beta \varphi -C_{\alpha \beta }^{\rho }J_{\rho }\text{cos}\beta \varphi \right)\text{sin}\beta \phi ]\\ & =\frac{\mu_{0}}{4\pi }\text{d}s\Sigma_{\alpha =0}^{\infty }\Sigma_{\beta =0}^{\alpha }\varepsilon_{\beta }C_{\alpha \beta }r^{\alpha }P_{\alpha }^{\beta }\left(\text{cos}\theta \right){\left(\begin{array}{c} C_{\alpha \beta }^{\varphi }J_{\varphi }cos\beta \varphi +C_{\alpha \beta }^{\rho }J_{\rho }sin\beta \varphi \\ C_{\alpha \beta }^{\varphi }J_{\varphi }sin\beta \varphi -C_{\alpha \beta }^{\rho }J_{\rho }cos\beta \varphi \end{array}\right)}^{T}\left(\begin{array}{c} \text{cos}\beta \phi \\ \text{sin}\beta \phi \end{array}\right). \end{array}$$ (18)

From Fig 2, *r*' = (*a* + *ρ*)^1/2^, sin*θ*' = *ρ*/*r*' and cos*θ*' = *a*/*r*'. Therefore, *C*_*αβ*_, $\;C_{\alpha \beta }^{\rho }$, and $C_{\alpha \beta }^{\varphi }$ are all functions of *ρ* for given *α* and *β*.

Eq (18) may be integrated over the whole BSC,

$$B_{z}=\frac{\mu_{0}}{4\pi }\Sigma_{\alpha =0}^{\infty }\Sigma_{\beta =0}^{\alpha }\varepsilon_{\beta }r^{\alpha }P_{\alpha }^{\beta }\left(\text{cos}\theta \right){\left(\begin{array}{c} {A^{\prime }}_{\alpha +1}^{\beta }\\ {B^{\prime }}_{\alpha +1}^{\beta } \end{array}\right)}^{T}\left(\begin{array}{c} \text{cos}\beta \phi \\ \text{sin}\beta \phi \end{array}\right),$$ (19)

where

$${A^{\prime }}_{\alpha +1}^{\beta }=\int_{0}^{\rho }\int_{0}^{2\pi }\left(C_{\alpha \beta }^{\varphi }J_{\varphi }\text{cos}\beta \varphi +C_{\alpha \beta }^{\rho }J_{\rho }\text{sin}\beta \varphi \right)C_{\alpha \beta }\rho \text{d}\varphi \text{d}\rho$$

and

$${B^{\prime }}_{\alpha +1}^{\beta }=\int_{0}^{\rho }\int_{0}^{2\pi }(C_{\alpha \beta }^{\varphi }J_{\varphi }\text{sin}\beta \varphi -C_{\alpha \beta }^{\rho }J_{\rho }\text{cos}\beta \varphi )C_{\alpha \beta }\rho \text{d}\varphi \text{d}\rho .$$

Eqs (10) and (19) have similar forms, both have the term $r^{\alpha }P_{\alpha }^{\beta }\left(\text{cos}\theta \right)\left(\begin{array}{c} \text{cos}\beta \phi \\ \text{sin}\beta \phi \end{array}\right),$ which indicates they should have the same harmonic fields, i.e., sinusoidal harmonic fields. Therefore, BSC-generated harmonic fields could eliminate the effects of high order harmonic fields from the main field (Eq (10)) by selecting an appropriate current distribution. The effect of current distribution is embodied in ${A^{\prime }}_{\alpha +1}^{\beta }$ and ${B^{\prime }}_{\alpha +1}^{\beta }$, which are discussed below.

The continuous current density, ***J***(*ρ*, *φ*), can be divided into a tangential component, *J*_*φ*_, and radial component, *J*_*ρ*_. ***J***(*ρ*, *φ*) satisfies continuous flow, i.e., **∇*J*** = 0. Let us introduce the stream function *S*(*ρ*, *φ*), such that

$$J_{\varphi }=-\frac{\partial S}{\partial \rho },J_{\rho }=\frac{\partial S}{\rho \partial \varphi }.$$ (20)

Then the stream function may be expressed as

$$S^{\pm }\left(\rho ,\varphi \right)={\left(\pm 1\right)}^{l+k}\text{cos}k\varphi \;\Sigma_{q=1}^{Q}U_{q}s_{q}\left(\rho \right),\;0\leq \rho \leq \rho_{max},$$ (21)

where *l* and *k* are the degree and order of coils, respectively; "±" represents the upper and lower planes, respectively; and the expansion basis, *s*_*q*_ (*ρ*), may be randomly chosen, e.g. *s*_*q*_ (*ρ*) = sin*qπρ*⁄*ρ*_*max*_. Eq (21) is a Cos stream function. The current density’s tangential component and radial component may be derived from Eqs (20) and (21),

$$\{\begin{array}{c} J_{\varphi }^{\pm }={\left(\pm 1\right)}^{l+k}\text{cos}k\varphi \;\Sigma_{q=1}^{Q}U_{q}j_{\varphi ,q}\left(\rho \right)\\ J_{\rho }^{\pm }={\left(\pm 1\right)}^{l+k}\text{sin}k\varphi \;\Sigma_{q=1}^{Q}U_{q}j_{\rho ,q}\left(\rho \right) \end{array},$$ (22)

where *U*_*q*_ is the current coefficient, *j*_*φ*,*q*_ (*ρ*) = −∂*s*_*q*_ (*ρ*)⁄∂*ρ*, and *j*_*ρ*,*q*_ (*ρ*) = −*ks*_*q*_ (*ρ*)⁄*ρ*.

Therefore, substituting Eqs (22) into (19),

$$\begin{array}{ll} {A^{\prime }}_{\alpha +1}^{\beta } & =\int_{0}^{\rho }\int_{0}^{2\pi }\left[(C_{\alpha \beta }^{\varphi +}J_{\varphi }^{+}+C_{\alpha \beta }^{\varphi -}J_{\varphi }^{-}\right)\text{cos}\beta \varphi +(C_{\alpha \beta }^{\rho +}J_{\rho }^{+}+C_{\alpha \beta }^{\rho -}J_{\rho }^{-})\text{sin}\beta \varphi ]C_{\alpha \beta }\rho \text{d}\varphi \text{d}\rho \\ & =\overset{Q}{\underset{q=1}{\sum }}U_{q}\int_{0}^{\rho_{max}}\int_{0}^{2\pi }\begin{array}{c} \{[C_{\alpha \beta }^{\varphi +}+{\left(-1\right)}^{l+k}C_{\alpha \beta }^{\varphi -}]j_{\varphi ,q}\text{cos}k\varphi \text{cos}\beta \varphi \\ +[C_{\alpha \beta }^{\rho +}+{\left(-1\right)}^{l+k}C_{\alpha \beta }^{\rho -}]j_{\rho ,q}\text{sin}k\varphi \text{sin}\beta \varphi \} \end{array}C_{\alpha \beta }\rho \text{d}\varphi \text{d}\rho , \end{array}$$ (23)

and

$$\begin{array}{ll} {B^{\prime }}_{\alpha +1}^{\beta } & =\int_{0}^{\rho }\int_{0}^{2\pi }\left[(C_{\alpha \beta }^{\varphi +}J_{\varphi }^{+}+C_{\alpha \beta }^{\varphi -}J_{\varphi }^{-}\right)\text{sin}\beta \varphi -(C_{\alpha \beta }^{\rho +}J_{\rho }^{+}+C_{\alpha \beta }^{\rho -}J_{\rho }^{-})\text{cos}\beta \varphi ]C_{\alpha \beta }\rho \text{d}\varphi \text{d}\rho \\ & =\overset{Q}{\underset{q=1}{\sum }}U_{q}\int_{0}^{\rho_{max}}\int_{0}^{2\pi }\begin{array}{c} \{[C_{\alpha \beta }^{\varphi +}+{\left(-1\right)}^{l+k}C_{\alpha \beta }^{\varphi -})]j_{\varphi ,q}\text{cos}k\varphi \text{sin}\beta \varphi \\ -[C_{\alpha \beta }^{\rho +}+{\left(-1\right)}^{l+k}C_{\alpha \beta }^{\rho -}]j_{\rho ,q}\text{sin}k\varphi \text{cos}\beta \varphi \} \end{array}C_{\alpha \beta }\rho \text{d}\varphi \text{d}\rho . \end{array}$$ (24)

Considering $\int_{0}^{2\pi }\text{cos}k\varphi \text{sin}\beta \varphi \text{d}\varphi =0$ and $\int_{0}^{2\pi }\text{sin}k\varphi \text{cos}\beta \varphi \text{d}\varphi =0$, Eq (24) becomes

$${B^{\prime }}_{\alpha +1}^{\beta }=0,$$ (25)

and $\int_{0}^{2\pi }\text{cos}k\varphi \text{cos}\beta \varphi \text{d}\varphi =2\pi \delta_{k\beta }/\varepsilon_{\beta }$ and $\int_{0}^{2\pi }\text{sin}k\varphi \text{sin}\beta \varphi \text{d}\varphi =(\varepsilon_{\beta }-1)\pi \delta_{k\beta }$, $C_{\alpha \beta }^{\varphi -}={\left(-1\right)}^{\alpha +\beta }C_{\alpha \beta }^{\varphi +}$, and $\;C_{\alpha \beta }^{\rho -}={\left(-1\right)}^{\alpha +\beta }C_{\alpha \beta }^{\rho +}$, because cos(*π* − *θ*') = −cos*θ*', sin(*π* − *θ*') = sin*θ*' and $P_{\alpha }^{\beta }\left(-\text{cos}\theta^{\prime }\right)=P_{\alpha }^{\beta }\left(\text{cos}\theta^{\prime }\right)$, Eq (23) becomes

$$\begin{array}{ll} {A^{\prime }}_{\alpha +1}^{\beta } & =\Sigma_{q=1}^{Q}U_{q}\int_{0}^{\rho_{max}}\begin{array}{c} \{[C_{\alpha \beta }^{\varphi +}+{\left(-1\right)}^{l+k+\alpha +\beta }C_{\alpha \beta }^{\varphi +}]j_{\varphi ,q}2\pi \delta_{k\beta }/\varepsilon_{\beta }\\ +[C_{\alpha \beta }^{\rho +}+{\left(-1\right)}^{l+k+\alpha +\beta }C_{\alpha \beta }^{\rho +}]j_{\rho ,q}(\varepsilon_{\beta }-1)\pi \delta_{k\beta }\} \end{array}C_{\alpha \beta }\rho \text{d}\rho \\ & =\left[1+{\left(-1\right)}^{l+k+\alpha +\beta }\right]\pi \delta_{k\beta }\Sigma_{q=1}^{Q}U_{q}\int_{0}^{\rho_{max}}[2C_{\alpha \beta }^{\varphi +}j_{\varphi ,q}/\varepsilon_{\beta }+C_{\alpha \beta }^{\rho +}j_{\rho ,q}(\varepsilon_{\beta }-1)]C_{\alpha \beta }\rho \text{d}\rho , \end{array}$$ (26)

where *δ*_*kβ*_ is the Kronecker function. For ${A^{\prime }}_{\alpha +1}^{\beta }\neq 0$, two conditions must be satisfied,

$$\{\begin{array}{c} \beta =k\\ \left(\alpha +\beta +l+k\right)\text{ mod }2=0 \end{array}.$$ (27)

Therefore, Eq (19) becomes

$$B_{z}=\frac{\mu_{0}}{4\pi }\Sigma_{\alpha =0}^{\infty }\Sigma_{\beta =0}^{\alpha }\varepsilon_{\beta }A^{\prime }{}_{\alpha +1}^{\beta }r^{\alpha }P_{\alpha }^{\beta }\left(\text{cos}\theta \right)\text{cos}\beta \phi ,$$ (28)

and BSCs can only generate the Cos harmonic fields from Eq (28), i.e.,

$$r^{\alpha }P_{\alpha }^{\beta }\left(\text{cos}\theta \right)\text{cos}\beta \phi .$$ (29)

Hence, Eq (28) is called the Cos *B*_*z*_ function, which is determined by the form of the stream function discussed further below.

If we change the stream function Eq (21) into the other form,

$$S^{\pm }\left(\rho ,\varphi \right)={\left(\pm 1\right)}^{l+k}\text{sin}k\varphi \;\Sigma_{q=1}^{Q}U_{q}s_{q}\left(\rho \right),\;0\leq \rho \leq \rho_{max},$$ (30)

which still satisfies continuous flow. Eq (30) is called a Sin stream function, and the corresponding current density may be expressed as

$$\{\begin{array}{c} J_{\varphi }^{\pm }={\left(\pm 1\right)}^{l+k}\text{sin}k\varphi \;\Sigma_{q=1}^{Q}U_{q}j_{\varphi ,q}^{\text{'}}\left(\rho \right)\\ J_{\rho }^{\pm }={\left(\pm 1\right)}^{l+k}\text{cos}k\varphi \;\Sigma_{q=1}^{Q}U_{q}j_{\rho ,q}^{\text{'}}\left(\rho \right) \end{array},$$ (31)

where *U*_*q*_ is still the current coefficient, $j_{\varphi ,q}^{\prime }\left(\rho \right)=j_{\varphi ,q}\left(\rho \right)=-\partial s_{q}\left(\rho \right)/\partial \rho$, and $j_{\rho ,q}^{\prime }\left(\rho \right)=-j_{\rho ,q}\left(\rho \right)=ks_{q}\left(\rho \right)/\rho$.

Substituting Eqs (31) into (19), and following the derivation above,

$${A^{\prime }}_{\alpha +1}^{\beta }=0,$$ (32)

and

$$\begin{array}{ll} {B^{\prime }}_{\alpha +1}^{\beta } & =\Sigma_{q=1}^{Q}U_{q}\int_{0}^{\rho_{max}}\begin{array}{c} \{[C_{\alpha \beta }^{\varphi +}+{\left(-1\right)}^{l+k+\alpha +\beta }C_{\alpha \beta }^{\varphi +}]j_{\varphi ,q}^{\prime }(\varepsilon_{\beta }-1)\pi \delta_{k\beta }\\ -[C_{\alpha \beta }^{\rho +}+{\left(-1\right)}^{l+k+\alpha +\beta }C_{\alpha \beta }^{\rho +}]j_{\rho ,q}^{\prime }2\pi \delta_{k\beta }/\varepsilon_{\beta }\} \end{array}C_{\alpha \beta }\rho \text{d}\rho \\ & =\left[1+{\left(-1\right)}^{l+k+\alpha +\beta }\right]\pi \delta_{k\beta }\Sigma_{q=1}^{Q}U_{q}\int_{0}^{\rho_{max}}\left[(\varepsilon_{\beta }-1\right)C_{\alpha \beta }^{\varphi +}j_{\varphi ,q}^{\prime }-2C_{\alpha \beta }^{\rho +}j_{\rho ,q}^{\prime }/\varepsilon_{\beta }]C_{\alpha \beta }\rho \text{d}\rho \\ & =\left[1+{\left(-1\right)}^{l+k+\alpha +\beta }\right]\pi \delta_{k\beta }\Sigma_{q=1}^{Q}U_{q}\int_{0}^{\rho_{max}}\left[2C_{\alpha \beta }^{\rho +}j_{\rho ,q}/\varepsilon_{\beta }+C_{\alpha \beta }^{\varphi +}j_{\varphi ,q}(\varepsilon_{\beta }-1\right)]C_{\alpha \beta }\rho \text{d}\rho . \end{array}$$ (33)

When ${B^{\prime }}_{\alpha +1}^{\beta }\neq 0$, Eq (27) must be satisfied, and similarly Eqs (26) and (33). Therefore, Eq (19) becomes

$$B_{z}=\frac{\mu_{0}}{4\pi }\Sigma_{\alpha =0}^{\infty }\Sigma_{\beta =0}^{\alpha }\varepsilon_{\beta }B^{\prime }{}_{\alpha +1}^{\beta }r^{\alpha }P_{\alpha }^{\beta }\left(\text{cos}\theta \right)\text{sin}\beta \phi ,$$ (34)

which is called a Sin*B*_*z*_ function.

which can only generate Sin harmonic fields, i.e.,

$$r^{\alpha }P_{\alpha }^{\beta }\left(\text{cos}\theta \right)\text{sin}\beta \phi .$$ (35)

Since Eqs (28) and (34) both meet the conditions of Eq (27), when *β* = *k*, the conclusion that the stream function type determines the *B*_*z*_ function type is evident by comparing Eqs (27) and (34) with Eqs (21) and (30). In other words, Sin stream functions can only generate Sin harmonic fields and the Cos stream functions can only generate Cos harmonic fields. If we want to generate corresponding higher harmonic fields while retaining uniform *B*_*z*0_ as mentioned above, the target harmonic field type must be first determined from Table 1. Eq (30) shows that the stream and harmonic functions must choose Cos function when *β* = *k* = 0. Eq (28) shows the results as [12]. However, the methodology in the current paper is more general, since it is valid for spherical harmonics that include Sin and Cos functions simultaneously, whereas [12] only presented Cos harmonic functions, with the generation of Sin harmonic functions only represented by rotating at specific angles. Although [12] conclusions were correct, they lacked a strict proof.

When *β* = *k* ≠ 0, *ε*_*β*_ = 2, and Eqs (26) and (33) have the same form, i.e. ${A^{\prime }}_{\alpha +1}^{\beta }={B^{\prime }}_{\alpha +1}^{\beta }$. Thus, Cos and Sin *B*_*z*_ functions would have the same coefficients. Comparing Eqs (28) and (34), the difference between them is just trigonometry on *ϕ*, i.e., cos*βϕ* and sin*βϕ*. Hence, by rotating the coils *π*/2*β* degrees clockwise, Cos harmonics are changed into Sin harmonics with the same *α* and *β*.

The condition *α*≥*β* should also be considered along with the two conditions of Eq (27),

$$\{\begin{array}{c} \beta =k\\ \alpha =\beta +\left(l+k\right)\text{mod}\;2+2\left(i-1\right) \end{array},$$ (36)

where *i* = 1,2,3,…*N*; and *N* represents the number of generated harmonic fields.

Eq (36) shows that the shim coil for a given degree *l* and order *k* can generate an infinite number of harmonic fields depending on the value of *i*. Hence, the fields generated by a coil with degree *l* and order *k* may be expressed as

$$B_{l}^{k}{}_{z}\left(i\right)=\frac{\mu_{0}}{4\pi }\varepsilon_{\beta }A^{\prime }{}_{\alpha \left(i\right)+1}^{k}r^{\alpha \left(i\right)}P_{\alpha \left(i\right)}^{k}\left(\text{cos}\theta \right)\text{cos}k\phi ,$$ (37)

and from Eq (26),

$$B_{l}^{k}{}_{z}\left(i\right)=\Sigma_{q=1}^{Q}U_{q}D\left(i,q\right)r^{\alpha \left(i\right)}P_{\alpha \left(i\right)}^{k}\left(\text{cos}\theta \right)\text{cos}k\phi ,$$ (38)

where

$$D\left(i,q\right)=\frac{\mu_{0}\varepsilon_{k}}{4}\int_{0}^{\rho_{max}}[2C_{\alpha \left(i\right)k}^{\varphi +}j_{\varphi ,q}/\varepsilon_{k}+C_{\alpha \left(i\right)k}^{\rho +}j_{\rho ,q}(\varepsilon_{k}-1)]C_{\alpha \left(i\right)k}\rho \text{d}\rho .$$ (39)

Eq (38) may be expressed in matrix form as

$$V_{\alpha }^{k}=DU,$$ (40)

where *U* = [*U*_1_, *U*_2_, *U*_3_ ⋯*U*_*Q*_]^T^, and $V_{\alpha }^{k}={\left[V_{\alpha \left(1\right)}^{k},\;V_{\alpha \left(2\right)}^{k},V_{\alpha \left(3\right)}^{k},\cdots V_{\alpha \left(N\right)}^{k}\right]}^{\text{T}}$. *D*(*i*, *q*) can be calculated from Eq (39), which is constant for the corresponding values of *i* and *q*.$V_{\alpha }^{k}$ is the coefficient matrix of harmonic fields.

The restricting condition,

$$V_{\alpha }^{k}=\delta_{l,\alpha \left(i\right)}V_{\alpha }^{k}=V_{l}^{k},$$ (41)

will make the coil only generate one specific type of harmonic field with *l* = *α*(*i*) while the other *N-1* types will not be generated. Values of degree *l* and order *k* may be given as targets in advance. The nonzero term’s value of $V_{l}^{k}$ may be arbitrarily chosen except zero, because $V_{l}^{k}$ is proportional to the current coefficient matrix, *U*, when *D* is constant, and the current coefficient matrix *U* can be solved. *U*’s magnification only changes the current magnitude, not the distribution. For example, when *l* = 1, *k* = 0, then *β* = 0 and *α* = 2*i* − 1 from Eq (36), and there should be *N* types of harmonic fields generated by the coil. However, from Eqs (41) and (29), only the $rP_{1}^{0}\left(\text{cos}\theta \right)$ harmonic field is built, i.e., the *Z* direction gradient field.

*S*(*ρ*, *φ*) may be obtained by substituting the solved matrix *U* into Eq (21). However, since it is continuous, it cannot be used directly for engineering applications. Therefore, *S*(*ρ*, *φ*) must be processed discretely before application.

*S*(*ρ*, *φ*) may be represented by some current-carrying coils, with the coil current, determined by

$$I_{0}=(I_{max}-I_{min})/M,$$ (42)

where *I*_*max*_ and *I*_*min*_ are the maximum and minimum values of *S*(*ρ*, *φ*), respectively; and *M* is the order of *S*(*ρ*, *φ*), which is set in advance. Therefore, the discrete *S*(*ρ*, *φ*) can be expressed as

$$S\left(\rho ,\varphi \right)=\left(j+0.5\right)I_{0}\;+\;I_{min},$$ (43)

where *j* = 0, 1, 2, 3⋯M − 1.

Eq (43) indicates that the current stream distribution function, *S*(*ρ*, *φ*), is expressed by the corresponding order contour lines.

## Design diagram and results for shim coils

**Fig 3:**
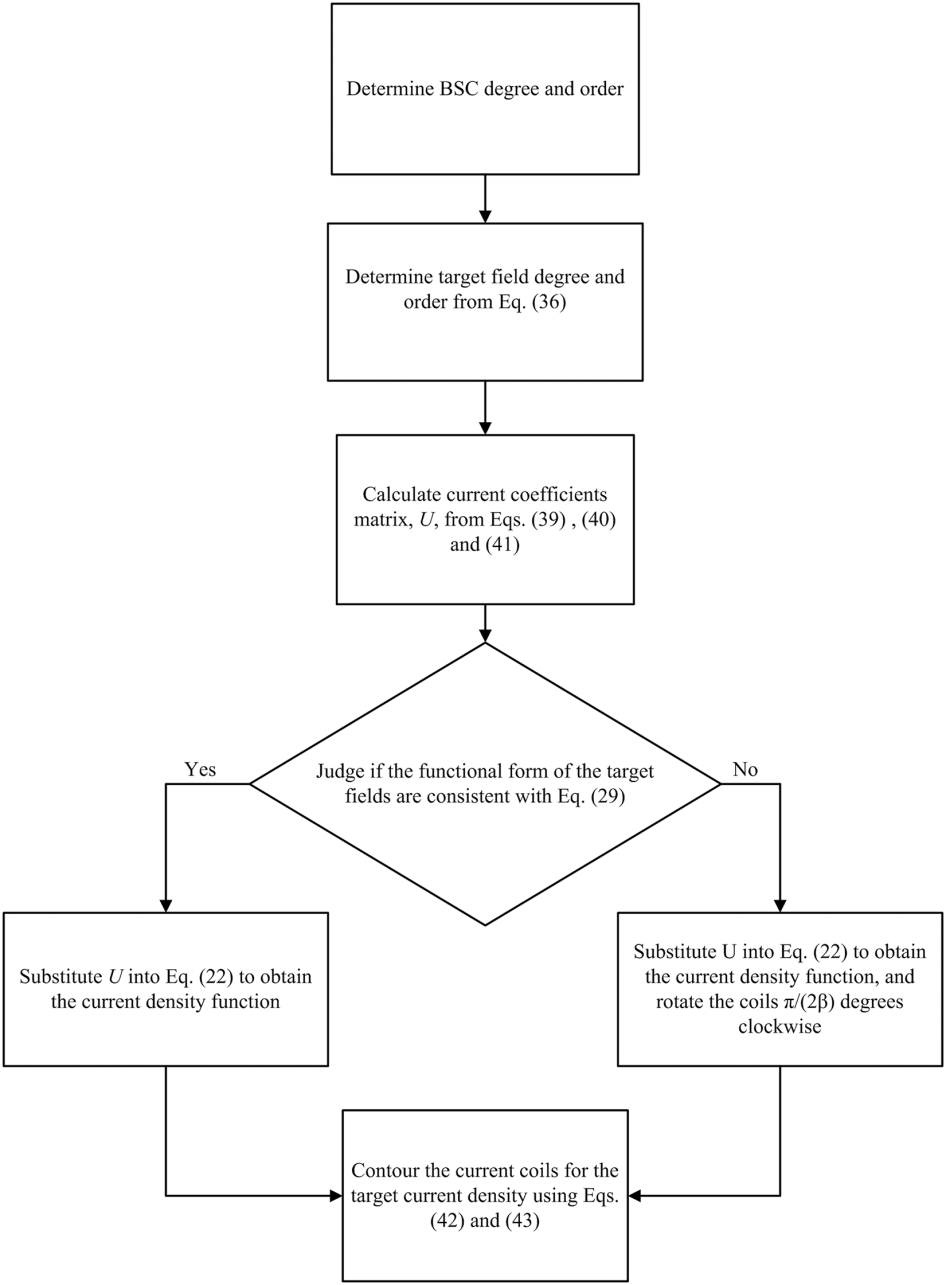
Flow diagram for biplanar shim coil design.

Fig 3 shows the shim coil design process based on the above methodology as a flow diagram. Several specific examples of target fields design processes are presented. Two parameters are required first: Q = 4 and *N* = 4 were used for all the design coils.

### Case 1

1. The stream function degree and order were set as *l* = 1 and *k* = 1, respectively.
2. The generated harmonic field’s degree and order were determined from Eq (36), i.e., *α* = 2*i* − 1 and *β* = *k* = 1.
3. Eqs (41) and (29) ensure that only one type of target harmonic field will be generated, i.e. $rP_{1}^{0}\left(\text{cos}\theta \right)\text{cos}\phi$ with *α* = 1, *β* = 1 and $V_{\alpha }^{k}={\left[V_{1}^{1},\;0,0,0\right]}^{\text{T}}$. From Table 1, *r*sin *θ* cos*ϕ* is the X type harmonic in the rectangular coordinate system.
4. The current coefficient matrix *U* was solved by Eq (40), and then the stream function *S*(*ρ*, *φ*) of generating X type harmonic field was obtained by substituting *U* into Eq (21).
5. The discrete *S*(*ρ*, *φ*) was calculated from Eqs (42) and (43), and is called an X coil.
6. The Y coil generating the Y harmonic was obtained by rotating the X coil *π* / 2 degrees clockwise based on the above conclusion.

### Case 2

1. The stream function degree and order were set as *l* = 2 and *k* = 1, respectively.
2. The degree generated harmonic field order was determined from Eq (36), i.e. *α* = 2*i* and *β* = *k* = 1.
3. Eqs (41) and (29) ensure one type of target harmonic field will be generated, i.e. $r^{2}P_{2}^{1}\left(\text{cos}\theta \right)\text{cos}\phi$ with *α* = 2, *β* = 1 and $V_{\alpha }^{k}={\left[V_{2}^{1},\;0,0,0\right]}^{\text{T}}$. From Table 1, *r*^2^cos *θ* sin *θ* cos*ϕ* is the XZ harmonic in the rectangular coordinate system.
4. The current coefficient matrix *U* was solved by Eq (40), and the stream function *S*(*ρ*, *φ*) generating the XZ harmonic field was obtained by substituting *U* into Eq (21).
5. The discrete *S*(*ρ*, *φ*) was calculated from Eqs (42) and (43), and is called an XZ coil.
6. The YZ coil generating the Y harmonic was obtained by rotating the XZ coil *π* / 2 degrees clockwise based on the above conclusion.

### Case 3

1. The stream function degree and order were set as *l* = 4 and *k* = 0, respectively.
2. The generated harmonic field degree and order were determined from Eq (36), i.e. *α* = 2(*i* − 1) and *β* = *k* = 0.
3. Eq (41) ensured only one type of target harmonic field will be generated, i.e. $r^{4}P_{4}^{0}\left(\text{cos}\theta \right)$ with *α* = 4, *β* = 0and $V_{\alpha }^{k}={\left[0,\;0,V_{4}^{0},0\right]}^{\text{T}}$. From Table 1, *r*^4^ (35cos^4^ θ − 30cos^2^ *θ* + 3) is the Z4 harmonic in the rectangular coordinate system.
4. The current coefficient matrix *U* was be solved by Eq (40), and the stream function *S*(*ρ*, *φ*) generating X harmonic fields was obtained by substituting *U* into Eq (21).
5. The discrete *S*(*ρ*, *φ*) was calculated from Eqs (42) and (43), and is called a Z4 coil.

**Fig 4:**
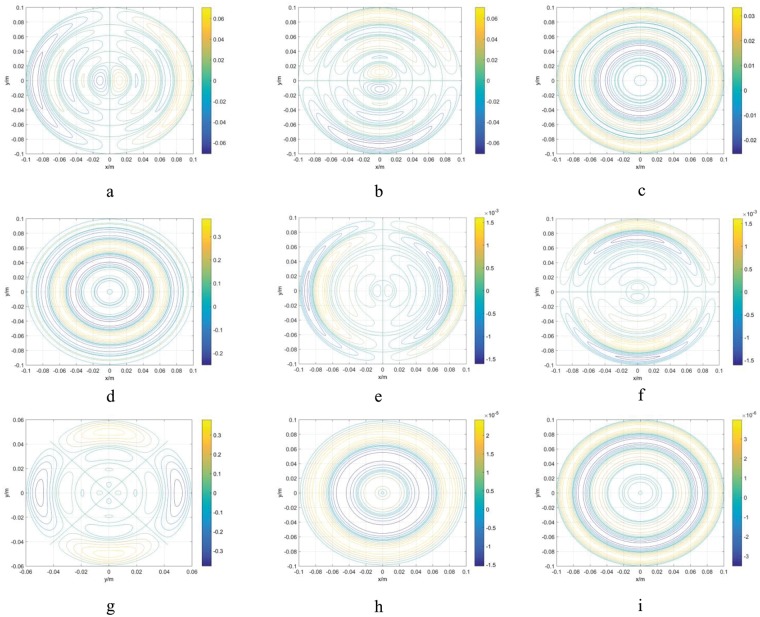
Calculated shim coils: (a) X, (b) Y, (c) Z, (d) Z2, (e) XZ, (f) YZ, (g) (X2-Y2), (h) Z3, and (i) Z4 harmonics, as detailed in Table 1.

Following the process of Fig 3, nine pairs of BSCs were calculated that generate X, Y, Z, Z^2^, XZ, YZ, X^2^-Y^2^, Z3 and Z4 harmonic fields (Table 1), as shown in Fig 4. The coils were used for shimming the main magnetic field of the high-performance relaxation analyzer. The gap between magnetic poles is 120 mm, i.e., *a* = 60mm, and the target field volume is Φ × H = 25.4 mm × 40 mm. Therefore, the maximum distance from the zero point is less than 60 mm, which meets the requirement of Eq (14).

**Fig 5:**
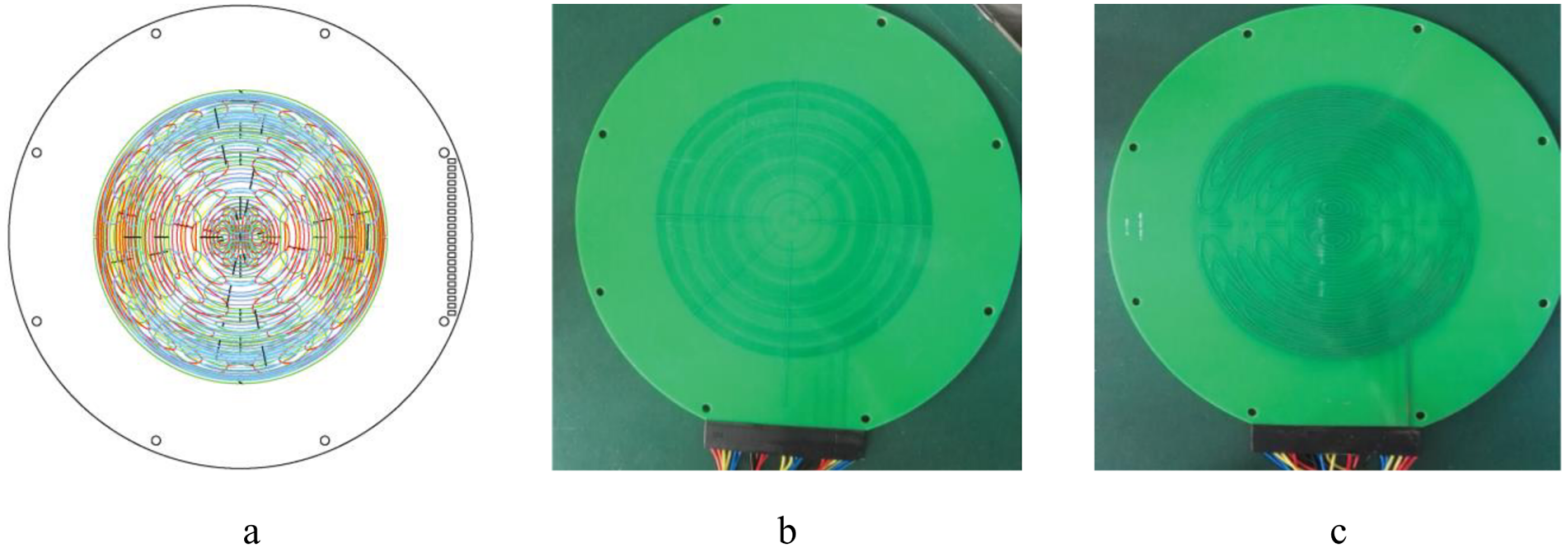
Example shim coil engineering drawing and PCBs. (a) the engineering drawing for the shim coils, (b) and (c) the multilayer PCBs.

Fig 4 shows different colored coils, where each color represents different current density strengths. During the design process, positive and negative current densities are represented by clockwise and anti-clockwise coils, respectively. Fig 5(a) shows the engineering drawing for the shim coils. The pair of printed circuit boards (PCBs) shown in Fig 5(b) and 5(c) are multilayer boards 2 mm thick. Each PCB for the nine shim coils was installed on the two poles of a permanent biplanar MRI in symmetry. A pair of coils generating the same harmonic in two PCBs were connected in series by wires. Thus, there were nine independent groups of constant current sources to power the nine BSC pairs, respectively.

## Experiments to test shimming effects

**Fig 6:**
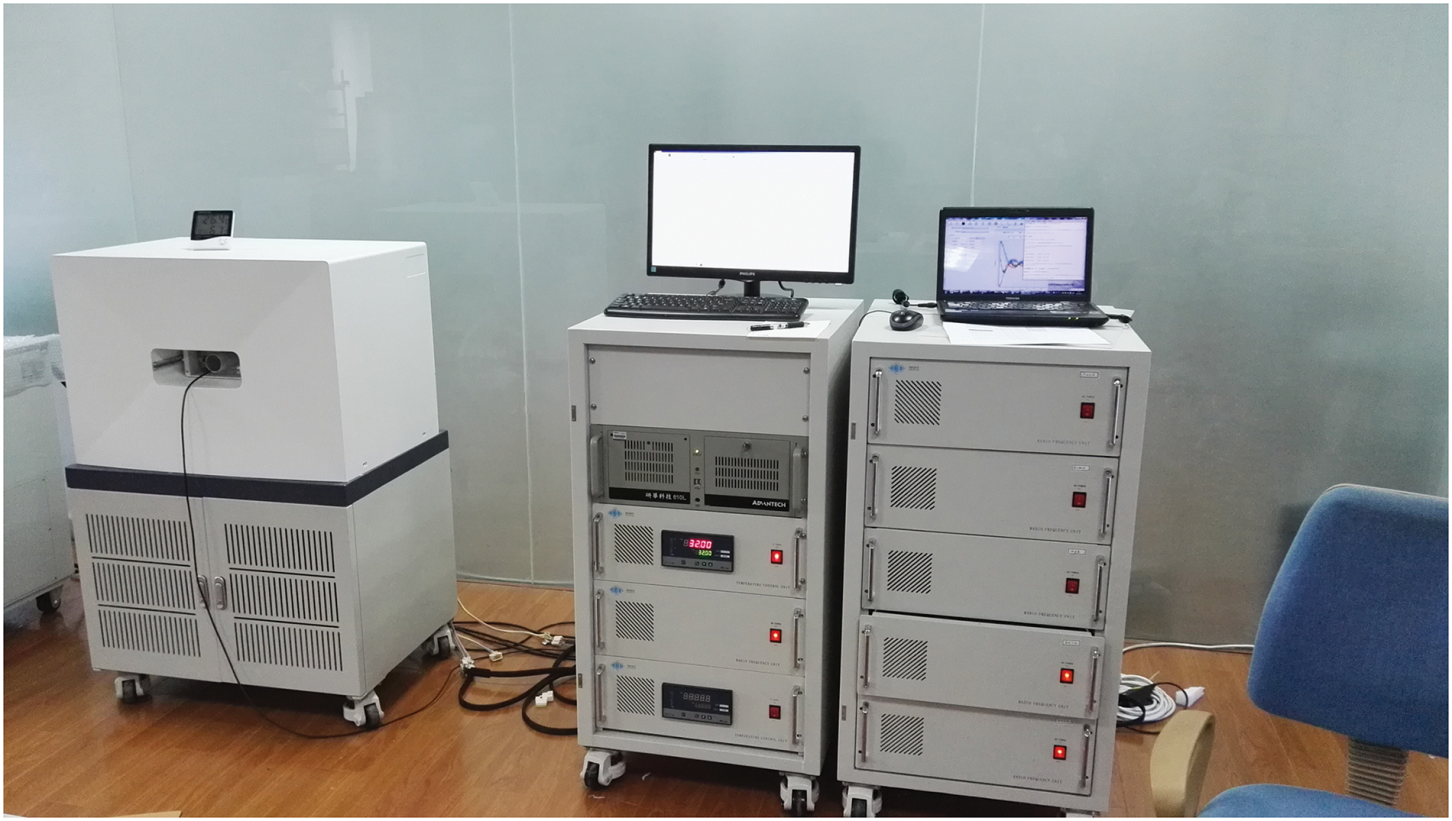
Shim coil effect test equipment.

An experiment was conducted to test the effects of the shim coils. The magnetic intensity of the biplanar permanent MRI = 0.5T, hence resonance frequency = 21.3 MHz for pure water. The adjustment order of shim coil current was Z, X, Y, XZ, YZ, Z^2^, X^2^-Y^2^, Z^3^, and Z^4^. The effect of shimming can be measured by comparing the free induced decay (FID) integral area before and after shimming. Once the current in the first BSC pair was adjusted to achieve maximum integral area, then the next BSC pair was adjusted, and so on to through all BSCs. BSC maximum current was 310 mA, and total thermal power < 10 W. Shim coil electric parameters are shown in Table 2, where the resistance terms represent the total resistance of a pair coil in series. Fig 6 shows the test equipment.

**Table 2:** Electric parameters for nine shim coils.

Shim Coil	Current (mA)	Resistance (Ω)	Thermal power (W)
**Z**	-205.0	**25.2**	**1.059**
**X**	256.0	**27.0**	**1.769**
**Y**	85.0	**27.0**	**0.195**
**XZ**	232.0	**23.0**	**1.238**
**YZ**	-304.0	**23.0**	**2.126**
**Z**^**2**^	10.0	**40.8**	**0.004**
**X**^**2**^**-Y**^**2**^	310.0	**30.8**	**2.960**
**Z**^**3**^	-40.0	**34.4**	**0.055**
**Z**^**4**^	**55.0**	**33.2**	**0.100**

**Fig 7:**
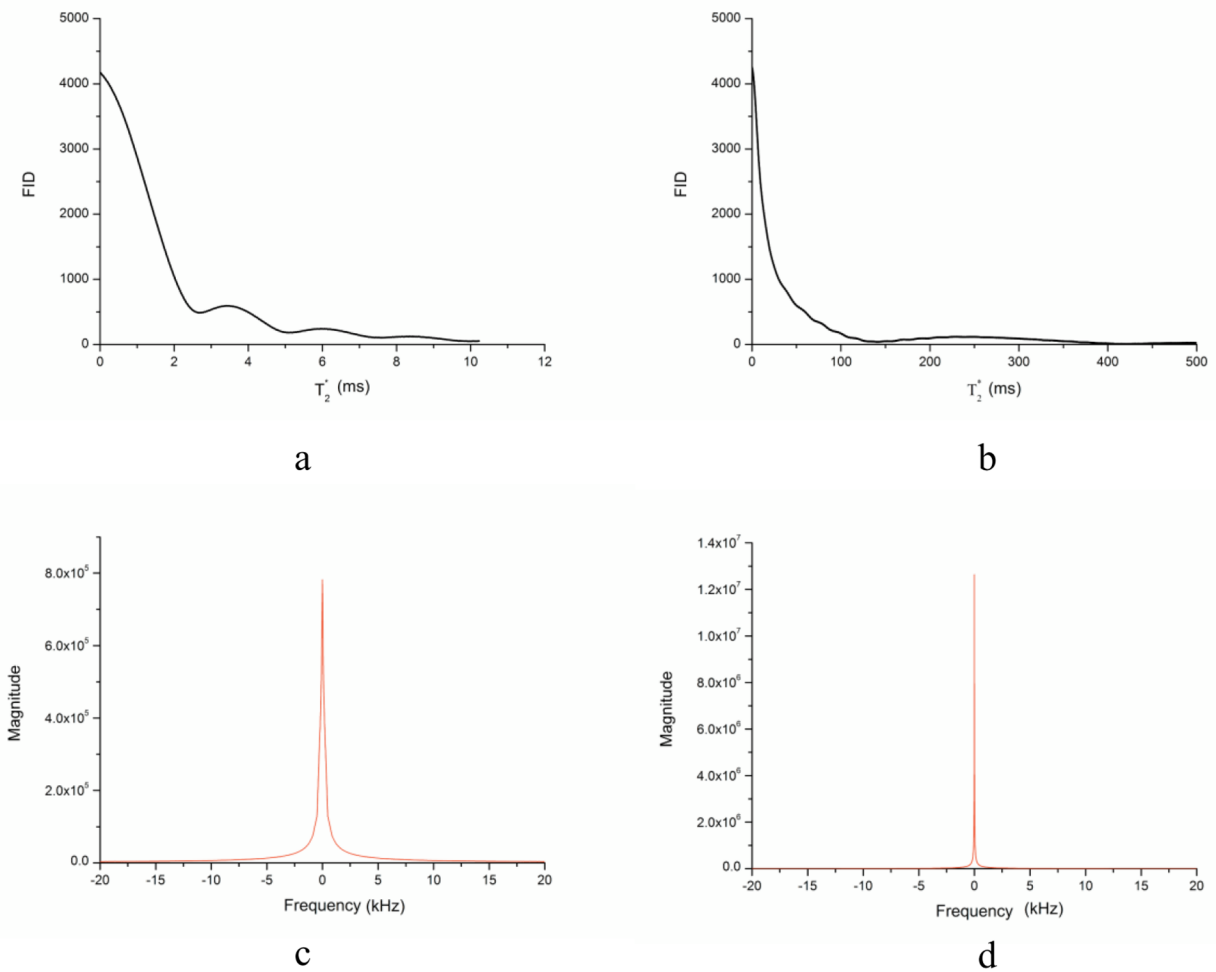
Free induced decay (FID) and frequency spectra (FFT) (a), (c) before and (b), (d) after shimming, respectively.

Magnetic dephasing caused by field inhomogeneity produces additional suppression of the signal. Hence, damping the magnitude of transverse magnetization is described by replacing the decay time, *T*_2_ with a modified time, $T_{2}^{*}$. Although qualitatively they have the same shape, the new envelope typically decays much faster in time. Decay time $T_{2}^{*}$ represents a combination of external field induced $T_{2}^{\prime }$ and thermodynamic *T*_2_ effects,

$$\frac{1}{T_{2}^{*}}=\frac{1}{T_{2}}+\frac{1}{T_{2}^{\prime }},$$ (44)

where $T_{2}^{\prime }$ is both machine and sample dependent[18]. Since the sample used in these experiments was pure water, and the sample’s effect on $T_{2}^{\prime }$ could be ignored, and hence, $T_{2}^{\prime }$ only depends on inhomogeneities of the main field in this case. Generally, $T_{2}^{\prime }$ dependence is used in FID measurements to determine if main field homogeneity is adequate. This method was also used in the experiment, and the Fourier transform of FID (data in S1 Table) are shown in Fig 7.

The shimming effect is clearly evident. As the FID integral area increases from 28,900 to 5,892,900, an approximately 20-fold increase, the full width at half maximum (FWHM) of the frequency spectrum decreased from 17.90 to 0.81 ppm, and the corresponding full width at 1/10 maximum (FWTM) decreased from 79.62 to 4.87 ppm.

Since thermal deformation of the shim coils will decrease the shimming effect, stability of the shimming effect was also considered, and estimated by measuring FWHM and FWTM every 10 minutes over 24 hours. The average change rate was approximately 0.50%, with a maximum rate of approximately 0.85%.

## Discussion and conclusion

The general expression for magnetic induction was derived from magnetic scalar potential and indicates that the field may be expanded as a series of harmonic fields, which is the basis of target field theory. Shimming aims to eliminate the effects of high order harmonic fields through reversed harmonic fields induced by the shim coils.

The harmonic fields contain Sin and Cos harmonic functions, induced by the corresponding Sin and Cos stream functions. The BSCs not only directly generated specific harmonic fields, but also generated other harmonic fields through specific operations.

The design process was shown diagrammatically, and nine BSCs were designed and fabricated based on the theory developed here, then installed in a biplanar permanent MRI. The shimming effect was very significant, which indicates that the theory and development approach is viable.

## Supporting information

S1 Table The raw FID data. (XLSX)
